# Effects of Open versus Laparoscopic Nephrectomy Techniques on Oxidative Stress Markers in Patients with Renal Cell Carcinoma

**DOI:** 10.1155/2013/438321

**Published:** 2013-02-24

**Authors:** Celestyna Mila-Kierzenkowska, Alina Woźniak, Tomasz Drewa, Bartosz Woźniak, Michał Szpinda, Ewa Krzyżyńska-Malinowska, Paweł Rajewski

**Affiliations:** ^1^The Chair of Medical Biology, Collegium Medicum of Nicolaus Copernicus University, Karłowicza 24, 85-092 Bydgoszcz, Poland; ^2^Department of Tissue Engineering, Collegium Medicum of Nicolaus Copernicus University, Karłowicza 24, 85-092 Bydgoszcz, Poland; ^3^Department of Neurosurgery, Stanisław Staszic Specialist Hospital, Rydygiera 1, 64-920 Piła, Poland; ^4^Department of Normal Anatomy, Collegium Medicum of Nicolaus Copernicus University, Karłowicza 24, 85-092 Bydgoszcz, Poland; ^5^Department of Nephrology and Internal Medicine, City Hospital, Szpitalna 19, 85-826 Bydgoszcz, Poland

## Abstract

The aim of the study was to determine the concentration of lipid peroxidation products, the activity of selected antioxidant and lysosomal enzymes, and protease inhibitor in patients with renal cell carcinoma who underwent radical nephrectomy. The studied group included 44 patients: 21 of them underwent open surgery, while 23 underwent laparoscopy. Blood samples were collected three times: before treatment and 12 hours and five days after nephrectomy. In blood of participants, the concentration of thiobarbituric acid reactive substances (TBARS), the activity of catalase (CAT), superoxide dismutase (SOD) and glutathione peroxidase (GPx), and the activity of acid phosphatase (AcP), arylsulfatase (ASA), cathepsin D (CTSD), and **α**_1_-antitrypsin (AAT) were assayed. No statistically significant differences in investigated parameters were found between studied groups. Moreover, TBARS concentration and CAT, SOD, and GPx activity were not altered in the course of both types of surgery. Five days after both open and laparoscopic nephrectomy techniques, AAT activity was higher than its activity 12 hours after the procedure. The obtained results suggest that laparoscopy may be used for nephrectomy as effectively as open surgery without creating greater oxidative stress. Reduced period of convalescence at patients treated with laparoscopy may be due to less severe response of acute-phase proteins.

## 1. Introduction

 Renal cell carcinoma (RCC) is the most common kidney cancer that arises from the cells of the renal tubule [1]. Despite of the fact that renal cell carcinoma is relatively rare compared with other cancers, its incidence is still increasing [2]. Cigarette smoking, obesity, hypertension, and/or related medications have been implicated as risk factors [3]. For localized RCC, the radical nephrectomy remains the mainstay of surgical treatment, however, techniques are being modified [4]. Since its introduction, laparoscopic radical nephrectomy has been established as a standard care for the surgical management of localized renal cell carcinoma [5, 6]. Laparoscopic surgery seems to have considerable advantages over open surgery, such as decreased blood loss, decreased postoperative pain, and less morbidity, as well as shorter hospital stay [7–9]. However, laparoscopy requires special skills using unfamiliar devices, and there is a limited pool of urologist, trained in laparoscopy [6, 9]. Moreover, the induction of pneumoperitoneum during laparoscopy procedure has several local and systemic effects [10]. Still very little is known about the effect of different nephrectomy techniques on antioxidant-oxidant balance and inflammatory markers in patients with RCC.

 An imbalance between the production of reactive oxygen species (ROS) and an ability of human system to detoxify them or easily repair the resulting damage of ROS may cause oxidative stress. The most often investigated process involved in reactive oxygen species generation in cells is lipid peroxidation. Lipid peroxidation products, such as thiobarbituric acid reactive substances (TBARS), change the capacity of biological membranes [11]. The final result of structural disorders of cellular constituents caused by ROS is the change of their function and even cell death [12]. Yet, human organism is endowed with a complex arsenal of antioxidant defense mechanisms protecting them from an increased ROS formation and their reactions with cell compounds. One of the defense mechanisms is based on antioxidant enzyme activity and includes superoxide dismutase (SOD), catalase (CAT), and glutathione peroxidase (GPx) [13]. Oxidative stress may also lead to some disturbances in lysosomal membranes, hence, provoking some changes in activity of lysosomal enzymes in blood serum. These enzymes are involved in housekeeping tasks such as turnover of intracellular proteins, antigen presentation, and bone remodeling, but they are also known to be found outside lysosomes in certain pathological conditions and participate in numerous diseases [14].

The aim of this study was to determine the effect of two different nephrectomy techniques on the concentration of lipid peroxidation products, the activity of main antioxidant enzymes, and the activity of selected lysosomal enzymes as well as protease inhibitor in patients with renal cell carcinoma who underwent open surgery and laparoscopy.

## 2. Materials and Methods

The study was conducted on the group of patients attending the Department of Urology of the Jan Biziel Regional Hospital in Bydgoszcz. The inclusion criteria were patients ≥18 years of age who underwent radical nephrectomy for localized renal cell carcinoma and agreed to voluntary participation in experiment. Patients suffering from any other disease were excluded from the study as the treatment may have an impact on antioxidants level in blood. Exclusion criteria were also cigarettes smoking and use of supplement of diet containing antioxidants. Blood samples were obtained from a total number of 44 subjects (24 men and 20 women) aged between 30 and 76 years. All of the patients underwent radical nephrectomy; however, two different techniques were used, and hence, the subjects were divided into two groups. The first group consists of 21 persons treated with open surgery, while the other included 23 subjects treated with laparoscopy.

Blood samples were collected from the basilic vein before nephrectomy and 12 hours and five days after both open surgery and laparoscopy. The Local Bioethical Committee at Collegium Medicum of the Nicolaus Copernicus University in Toruń agreement was obtained, and all subjects had given their written informed consent. 

Thiobarbituric acid reactive substances level was determined in blood plasma and erythrocytes according to Buege and Aust [15] method in the Esterbauer and Cheesman modification [16]. The method involves creation of coloured complex between lipid peroxidation products and thiobarbituric acid at the temperature of 100°C and in acidic environment. The maximum absorption of that complex occurs at a wavelength of 532 nm. The main product of lipid peroxidation reacting with thiobarbituric acid is malondialdehyde (MDA), and, therefore, the level of TBARS in plasma was expressed as nmol of MDA/mL and in the erythrocytes as nmol of MDA/gHb.

The activity of antioxidant enzymes was measured in erythrocytes. Beers and Sizer [17] method was used to assay catalase activity. This method is based on measurement of absorbance decrease of hydrogen peroxide, which is decomposed by catalase, measured at a wavelength of 240 nm. CAT activity was expressed as 10^4^ IU/gHb. Superoxide dismutase activity was performed by Misra and Fridovich method [18]. This procedure is based on SOD impeding the reaction of autooxidation of adrenaline to adrenochrome in an alkaline environment. SOD activity was expressed as U/gHb. Glutathione peroxidase activity was determined according to Paglia and Valentine [19] by a method based on the measurement of changes in absorbance at a wavelength of 340 nm, caused by oxidation of reduced nicotinamide adenine dinucleotide phosphate (NADPH). NADPH is a coenzyme of reduction of glutathione disulfide. The obtained oxidized glutathione is a product of reaction catalysed by glutathione peroxidase. Activity of GPx was expressed as U/gHb. 

The activity of lysosomal enzymes and protease inhibitor was measured in the blood serum. The activity of acid phosphatase was determined by means of Bessey's method [20]. The activity measure was the quantity of p-nitrophenol generated during enzymatic hydrolysis of 4-nitrophenylphosphate disodium salt used as a substrate. Roy's method modified by Błeszyński [21] was used to assay arylsulfatase activity. The substrate employed here was 4-nitrocatechol sulfate (4-NCS), and the measure recorded was the quantity of 4-nitrocatechol released during enzymatic hydrolysis. Cathepsin D activity was determined using the Anson method [22] based on measurement of tyrosine quantity released during hydrolysis of haemoglobin by CTSD. The activity of *α*_1_-antitrypsin was determined according to Eriksson [23]. This procedure relies on the evaluation of the level of trypsin inhibited by AAT present in 1 mL of blood serum.

All results were statistically analysed by means of factorial repeated-measures ANOVA test with post hoc analysis (Tukey's range test). Before running ANOVA, model assumptions were also tested (Shapiro-Wilk test for normality and Box's Test for homogeneity of covariance). The correlation coefficients (*r*) between parameters for an evaluation of relationships were also estimated. Changes with a level of significance *P* < 0.05 were regarded as statistically significant. 

## 3. Results

The concentration of TBARS both in blood plasma and in erythrocytes of patients with renal cell carcinoma after nephrectomy was not altered in patients who underwent either open or laparoscopic surgery as compared to the value before the surgical treatment (Table 1). There were also no statistically significant differences in thiobarbituric acid reactive substances concentration between the two groups of RCC patients. However, some increasing tendency in TBARS levels was noticed 12 hours and 5 days after the surgery as compared to the value before the intervention.

 Considering the activity of catalase, superoxide dismutase, and glutathione peroxidase in investigated groups of patients, no statistically significant changes were found as a result of radical nephrectomy. There were also no statistically significant differences between patients undergoing open surgery versus laparoscopy (Table 1). Yet, some statistically significant correlations between studied antioxidant enzymes were found. Before the treatment, there was a positive correlation (*r* = 0.52, *P* < 0.05) between CAT and SOD activity in group of patients treated with open surgery and negative correlation (*r* = −0.63, *P* < 0.01) between CAT activity and TBARS level in erythrocytes in patients before laparoscopy. In patients treated with laparoscopy 12 hours after the nephrectomy, positive correlation (*r* = 0.50, *P* < 0.05) was revealed between CAT and GPx activity. 

 No statistically significant differences were found in activity of investigated lysosomal enzymes and protease inhibitor between the patients treated with open surgery as compared to patients who were subjected to laparoscopy. However, some changes in their activity were found as a consequence of surgical treatment. The pattern of changes was similar in both groups of patients, but some differences were noticed. The activity of arylsulfatase decreased after the nephrectomy in comparison to the value before the intervention (Table 2). In patients treated with open surgery, it decreased by about 26% (*P* < 0.05) 12 hours and by about 22% (*P* < 0.05) 5 days after the nephrectomy. In subjects who underwent laparoscopy, it decreased even much more, by about 37% (*P* < 0.001) and 45% (*P* < 0.001) 12 hours and 5 days after nephrectomy, respectively. In turn, CTSD activity in both statistically significant groups increased as a result of surgical treatment (Table 2). At patients treated with open surgery, CTSD activity 12 hours after the procedure was 77% higher (*P* < 0.05), while 5 days after surgery 89% higher (*P* < 0.01) than before the treatment. Twelve hours and 5 days after laparoscopy, CTSD activity was 61% (*P* < 0.01) and 55% higher (*P* < 0.05), respectively. Comparing the activity of protease inhibitor in the course of treatment, its activity 12 hours after both techniques of nephrectomy decreased, but this was statistically insignificant. Five days after the treatment, AAT activity statistically significantly increased as compared to the value 12 hours after the treatment (Table 2). At patients subjected to open surgery, AAT activity was then 84% higher (*P* < 0.001), while at subjects treated with laparoscopy, it was 51% higher (*P* < 0.05). There were no statistically significant changes in AcP activity after open surgery or laparoscopy (Table 2).

 Considering correlation coefficients between all studied lysosomal enzymes and protease inhibitor, positive correlation was revealed between ASA and AcP activity (*r* = 0.49, *P* < 0.05) and between ASA and AAT activity (*r* = 0.66, *P* < 0.01) and negative correlation between CTSD and ASA activity (*r* = −0.78, *P* < 0.001) at patients treated with open surgery 5 days after the treatment. At patients subjected to laparoscopy, negative correlation between CTSD and ASA activity was found both 12 hours (*r* = −0.62, *P* < 0.05) and 5 days after (*r* = −0.81, *P* < 0.001) the nephrectomy. 

**Figure 1:**
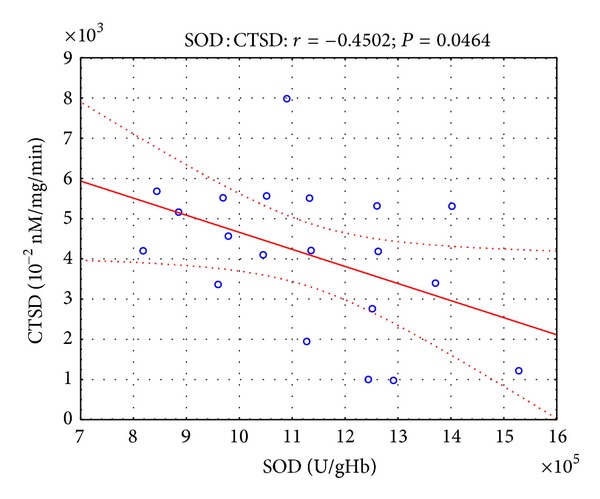
Linear regression of superoxide dismutase (SOD) activity versus cathepsin D (CTSD) activity, at patients with renal cell carcinoma, 12 hours after open surgery nephrectomy.

**Figure 2:**
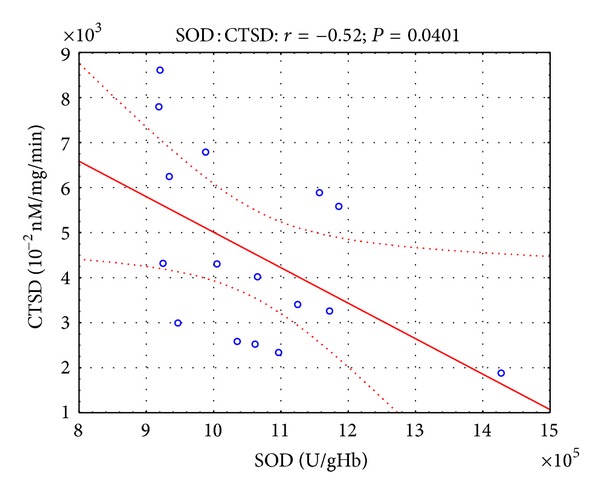
Linear regression of superoxide dismutase (SOD) activity versus cathepsin D (CTSD) activity, at patients with renal cell carcinoma, 12 hours after laparoscopic nephrectomy.

**Figure 3:**
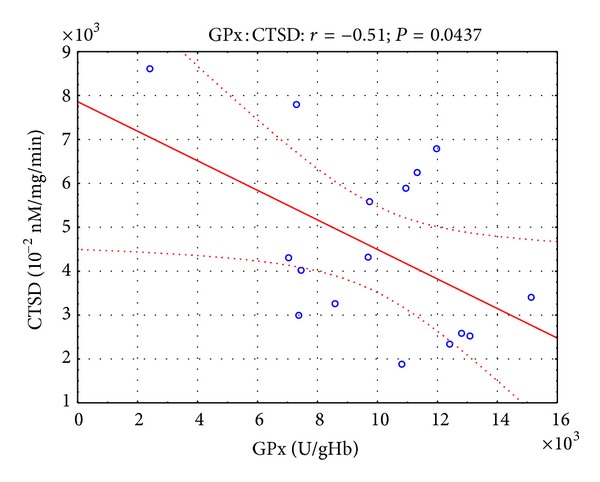
Linear regression of glutathione peroxidase (GPx) activity versus cathepsin D (CTSD) activity, at patients with renal cell carcinoma, 12 hours after laparoscopic nephrectomy.

**Figure 4:**
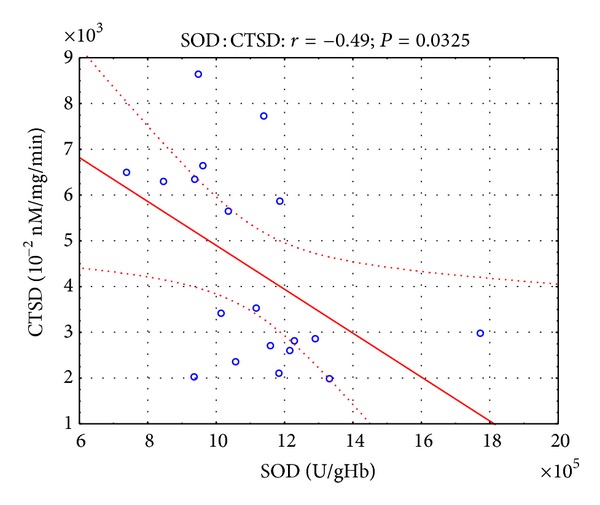
Linear regression of superoxide dismutase (SOD) activity versus cathepsin D (CTSD) activity, at patients with renal cell carcinoma, 5 days after open surgery nephrectomy.

**Figure 5:**
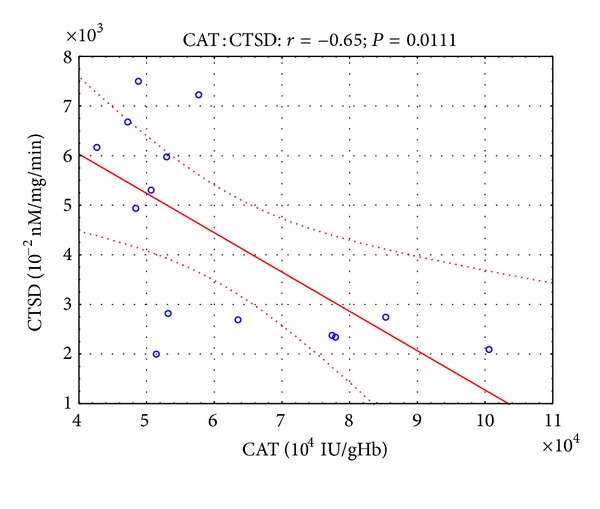
Linear regression of catalase (CAT) activity versus cathepsin D (CTSD) activity, at patients with renal cell carcinoma, 5 days after laparoscopic nephrectomy.

Moreover, in the presented study, some correlations were found between parameters of oxidative stress and lysosomal enzymes. Twelve hours after the open surgery, negative correlation (Figure 1) was observed between SOD and CTSD activity (*r* = −0.45, *P* < 0.05), while 12 hours after the laparoscopy, negative correlation (Figures 2 and 3) was revealed between SOD and CTSD activity (*r* = −0.52, *P* < 0.05) as well as between GPx and CTSD activity (*r* = −0.51, *P* < 0.05). Five days after the surgical treatment at patients subjected to open surgery, statistically significant negative correlation (Figure 4) was observed between SOD and CTSD activity (*r* = −0.49,   *P* < 0.05). In a group of patients who underwent laparoscopy, 5 days after the intervention, statistically significant negative correlation (Figure 5) was observed between CAT and CTSD activity (*r* = −0.65, *P* < 0.05), as well as positive correlation between SOD and ASA activity (*r* = 0.69, *P* < 0.01). Moreover, positive correlation between TBARS_plasma_ level and AAT activity (*r* = 0.59, *P* < 0.05) was found. 

## 4. Discussion

 Laparoscopic surgery has become one of the most important and popular technique in general surgery and is the procedure of choice for almost all types of abdominal operations, because of its advantages over open surgery [10]. Although it is technically demanding, laparoscopy provides RCC patients improved quality of life during recovery period with decreased analgetic requirements, fewer complications, and more rapid convalescence [24, 25]. However, there are some reports about higher in-hospital mortality and more common failure to rescue after laparoscopy probably due to poor experience of surgeons and hospitals [24, 25]. Despite all the advantages, laparoscopic surgery can have several local and systemic consequences related to the induction of pneumoperitoneum used to facilitate the visual field. Insufflation of carbon dioxide into peritoneal cavity leads to alterations in acid-base balance, blood gases, and cardiovascular and pulmonary physiology [26] and causes reversible renal dysfunction [27, 28]. The effects of pneumoperitoneum depend on many factors like the pressure level and gas used [29]. For example, helium seems to limit postoperative oxidative response following laparoscopy as higher MDA and carbonyl responses and sulfydryl consumption were revealed after CO_2_ insufflation compared with helium [30].

Recently, some clinical and experimental studies demonstrate that creating a pneumoperitoneum results in oxidative stress. Laparoscopy may among others alter the production of reactive oxygen species because of the effect of carbon dioxide on peroxynitrite metabolism [31]. Oxidative stress during surgical injury is also due to ischemia/reperfusion injury. During laparoscopy, increase of intra-abdominal pressure caused by pneumoperitoneum may cause splanchnic ischemia followed by reperfusion due to deflation [32]. An increase in lipid peroxidation products during the immediate postoperative period and postoperative decrease in endogenous antioxidants after laparoscopy, but not after open cholecystectomy, were shown by Glantzounis et al. [32]. The authors suggest that free radicals are generated at the end of laparoscopic procedure, possibly as a result of ischemia-reperfusion phenomenon induced by pneumoperitoneum. Increased oxidative stress due to pneumoperitoneum was also observed during laparoscopic donor nephrectomy, both in donor and remaining kidneys [25].

In our study, no statistically significant changes in the level of lipid peroxidation products and the activity of antioxidant enzymes were revealed both 12 hours and 5 days after open surgery or laparoscopy. There were also no differences in parameters of oxidative stress between patients treated with different nephrectomy techniques. The literature data about the effect of open and laparoscopic surgery techniques on parameters of oxidative stress are unequivocal. Gianotti et al. [33] demonstrated that both laparoscopy and open colon surgery may cause oxidative damage during mesentery traction and immediately after the end of operation. Bukan et al. [34] showed that both open and laparoscopic cholecystectomy techniques caused an increased oxidative stress; however, laparoscopy induces less significantly oxidative stress than open surgery. The others concluded that cholecystectomy, either open or laparoscopic, caused only moderate oxidative stress [31]. SOD activity and total antioxidant status was not changed after both procedures, while endogenous lipid peroxide level was higher onday 7 after intervention. Moreover, they found that the level of oxidized low density lipoproteins was higher after surgery, but only after open cholecystectomy. On the other hand, comparing open donor nephrectomy, laparoscopic donor nephrectomy, and retroperitoneoscopic donor nephrectomy, no differences were detected in oxidative stress markers in renal tissue samples [35]. 

Despite the lack of changes in oxidative stress markers after the surgical treatment, some statistically significant changes in ASA activity in serum of RCC patients after nephrectomy were found. However, it is known that this enzyme is a member of family of sulfatases activated by the oxidation of cysteine to formylglycine [36]. In turn, formylglycine-generating enzyme is inhibited by oxidation of its cysteine due to increased ROS generation [37]. Hence, it is possible that decrease in ASA activity observed in our study after nephrectomy is a consequence of presence of limiting factor for its activity related to increased generation of reactive oxygen species after the surgical treatment. 

Comparing open and laparoscopic nephrectomy techniques no statistically significant differences were found in cathepsin D activity; yet, after both procedures, its activity increased relevantly. Among the lysosomal hydrolases, cathepsins play major role in cellular proteolysis [38]. Originally, cathepsins were believed to participate exclusively in terminal protein degradation during necrotic and autophagic death. Nowadays, it is well established that those enzymes execute numerous specific functions participating in important physiological processes [39]. The increase in CTSD activity in blood serum is probably due to its release into cytosol after labilization of lysosomal membrane. Although the mechanism of lysosomal membrane destabilization is still poorly understood, the list of agents able to destabilize the membrane is very comprehensive and includes reactive oxygen species, which disturb the membranes by peroxidation of their lipids [40]. No significant statistically changes in antioxidant enzymes activity and lipid peroxidation level as a result of radical nephrectomy were found in this paper, but some statistically significant negative correlations between antioxidant enzymes and cathepsin D activity were revealed after surgical treatment in both groups of patients. This may suggest insufficient antioxidant defense after the surgery, which may lead to some oxidative damage, like lysosomes disruption. The confirmation of increased level of reactive oxygen species generation after the nephrectomy may be the increasing tendency in TBARS levels observed in this paper. Accumulating data suggest that ROSs not only act as damaging entitles, but also may carry important beneficial functions [41]. Reactive oxygen species occurring after the surgery may play a possible role in the healing of laparotomic wound [31]. 

The main way in which cathepsins activity is regulated is by interaction with their endogenous protease inhibitors [42]. In human plasma, serine protease inhibitors represent about 10% of the total protein, of which 70% is *α*_1_-antitrypsin [43]. AAT is nowadays considered as one of the acute-phase proteins as its normal plasma level increases 3-to 5-fold under variety of physiological and pathological conditions like stress, infections, and inflammation [44]. In present study, 12 hours after nephrectomy, the activity of *α*_1_-antitrypsin statistically insignificantly decreased, but 5 days after the procedure, its activity was higher than before treatment and 12 hours after the treatment. An increasing amount of evidences suggest that AAT possesses not only the ability to inhibit proteases, but also to exert anti-inflammatory and tissue protective effects independent of protease inhibition [45]. AAT in concert with other proteins is probably involved in protecting extracellular spaces from protein misfolding and precipitation especially under stress conditions [46]. Despite the fact that there were no statistically significant differences between patients from both studied groups, it can be noticed that the rise of AAT activity was higher after open surgery, which may suggest more intense inflammatory reaction than after laparoscopic procedure. Those results are in agreement with some research which compared inflammatory response in open and laparoscopic cholecystectomy techniques [47–49]. They found more significant increase in acute-phase inflammatory markers, such as *α*_1_-antitrypsin, after open surgery than after laparoscopy. They postulate that laparoscopic cholecystectomy, which is related to less tissue damage, provokes less intense stress response. Gál et al. [50] also showed that both open and laparoscopic procedures induced changes of acute-phase proteins level, free radical mediated reactions, and neutrophil functions. However, laparoscopy induces a significantly less intense response in these parameters. Considering the inflammatory response after open versus laparoscopic nephrectomy techniques, less intense surgical trauma-induced immune dysfunction was found after laparoscopy [51]. 

## 5. Conclusions

Our study demonstrates that neither open nor laparoscopic radical nephrectomy has effect on postoperative level of lipid peroxidation products and on activity of antioxidant enzymes in blood of RCC patients, and both techniques may cause only moderate changes in oxidant-antioxidant balance. Therefore, we believe that laparoscopy may be used for radical nephrectomy as effectively as open surgery without creating oxidative stress. Moreover, we have observed that the response of *α*_1_-antitrypsin is more severe after open surgery than after laparoscopy in studied RCC patients. The attenuation of acute-phase response may explain the reduced period of convalescence at patients treated with laparoscopy. 

## Figures and Tables

**Table 1:** Lipid peroxidation products level and antioxidant enzymes activity in patients with renal cell carcinoma before and after radical nephrectomy by open surgery and laparoscopy.

	Open radical nephrectomy (*n* = 21)		
	Before surgery	12 hours after surgery	5 days after surgery
TBARS_plasma_ (nmol MDA/mL)	0.51 ± 0.14	0.53 ± 0.12	0.55 ± 0.14
TBARS_erythrocytes_ (nmol MDA/gHb)	37.6 ± 13.2	39.0 ± 18.2	38.6 ± 14.5
CAT (10^4^ IU/gHb)	61.4 ± 18.6	67.2 ± 27.0	61.4 ± 19.6
SOD (U/gHb)	1167.6 ± 345.4	1132.5 ± 193.3	1110.3 ± 222.9
GPx (U/gHb)	9.0 ± 3.7	8.8 ± 3.2	7.8 ± 3.5

	Laparoscopic radical nephrectomy (*n* = 23)		
	Before laparoscopy	12 hours after laparoscopy	5 days after laparoscopy

TBARS_plasma_ (nmol MDA/mL)	0.45 ± 0.11	0.56 ± 0.13	0.56 ± 0.18
TBARS_erythrocytes_ (nmol MDA/gHb)	32.6 ± 19.2	40.1 ± 12.6	35.4 ± 12.9
CAT (10^4^ IU/gHb)	74.7 ± 32.3	55.8 ± 15.9	61.3 ± 17.3
SOD (U/gHb)	1161.0 ± 298.2	1060.3 ± 135.1	1011.7 ± 131.3
GPx (U/gHb)	6.9 ± 3.9	9.9 ± 3.1	6.5 ± 2.9

No statistically significant differences between open surgery and laparoscopy.

TBARS: thiobarbituric acid reactive substances; CAT: catalase; SOD: superoxide dismutase; GPx: glutathione peroxidase.

**Table 2:** Lysosomal enzymes and protease inhibitor activity in patients with renal cell carcinoma before and after radical nephrectomy by open surgery and laparoscopy.

	Open radical nephrectomy (*n* = 21)		
	Before surgery	12 hours after surgery	5 days after surgery
AcP (10^−2^ nM p-nitrophenol/mg protein/min)	1.87 ± 0.78	2.46 ± 0.89	2.22 ± 0.99
ASA (10^−3^ nM NCS/mg protein/min)	2.13 ± 0.46	1.58 ± 0.59^a^	1.66 ± 0.68^a^
CTSD (10^−2^ nM tyrosine/mg protein/min)	2.31 ± 1.19	4.10 ± 1.82^a^	4.37 ± 2.17^aa^
AAT (mg trypsin/mL serum)	0.62 ± 0.2	0.43 ± 0.2	0.79 ± 0.2^bbb^

	Laparoscopic radical nephrectomy (*n* = 23)		
	Before laparoscopy	12 hours after laparoscopy	5 days after laparoscopy

AcP (10^−2^ nM p-nitrophenol/mg protein/min)	2.07 ± 0.86	1.62 ± 0.79	1.61 ± 0.58
ASA (10^−3^ nM NCS/mg protein/min)	2.22 ± 0.36	1.40 ± 0.72^ccc^	1.23 ± 0.51^ccc^
CTSD (10^−2^ nM tyrosine/mg protein/min)	2.81 ± 0.97	4.53 ± 2.01^cc^	4.35 ± 2.10^c^
AAT (mg trypsin/mL serum)	0.61 ± 0.2	0.49 ± 0.2	0.74 ± 0.3^d^

No statistically significant differences between open surgery and laparoscopy.

Statistically significant differences: versus before open surgery: ^a^ *P* < 0.05, ^aa^ *P* < 0.01; versus 12 hours after open surgery: ^bbb^ *P* < 0.001; versus before laparoscopy: ^c^ *P* < 0.05 and ^cc^ *P* < 0.01 and ^ccc^ *P* < 0.001; versus 12 hours after laparoscopy: ^d^ *P* < 0.05.

AcP: acid phosphatase; AAT: *α*_1_-antitrypsin; ASA: arylsulphatase; CTSD: cathepsin D.
